# Supervisors’ Active-Empathetic Listening as an Important Antecedent of Work Engagement

**DOI:** 10.3390/ijerph17217976

**Published:** 2020-10-30

**Authors:** Inga Jona Jonsdottir, Kari Kristinsson

**Affiliations:** School of Business, University of Iceland, 101 Reykjavik, Iceland; karik@hi.is

**Keywords:** active-empathetic listening, social support, supervisor, work engagement, work-related well-being

## Abstract

Social support from supervisors is a job resource that has been found to be an important antecedent to work engagement. However, there is a knowledge gap in understanding one of the key features of social support—i.e., supervisors’ active-empathetic listening—and its relation to employees’ work engagement. To bridge this gap, this study explores how supervisors’ active-empathetic listening is associated with employees’ work engagement. Using a national representative sample (*N* = 548), the results show that supervisors’ active-empathetic listening has a significant positive relationship with employee work engagement. Additionally, we show that active-empathetic listening does not affect all three dimensions of work engagement equally, with dedication being the most affected by supervisors’ active-empathetic listening. We argue that supportive leadership which uses conscious and active listening-centred communication is highly significant for employees’ work engagement. Therefore, we suggest that organisations experiment in training their supervisors in active-empathetic listening as part of a broader strategy to increase employees’ engagement at work.

## 1. Introduction

Work engagement is a positive, fulfilling, and emotional state of mind associated with work. Most scholars agree that work engagement is characterised by a high level of energy and strong identification with one’s work (see e.g., [1,2,3,4]). Several studies have provided empirical evidence that high levels of work engagement are positively associated with job satisfaction (e.g., [5,6,7]) and low turnover intention (e.g., [8,9,10]). Research findings also indicate that work engagement is a predictor of high job performance [1,11], client satisfaction [1], and customer loyalty [12]. In addition, work engagement has been negatively associated with burnout [8,13]. 

According to Bakker and Demerouti [14], Halbesleben [15], and Bakker et al. [2], previous research has consistently confirmed the positive relationship between various job resources, such as social support from supervisors, work engagement, and positive work-related outcomes (see also, e.g., [3,5,7,16]). Social support is a job resource that has been found to be an important antecedent of work engagement [3,5,6,7,8,13,17]. Workplace social support refers to employees’ interaction and relationships with supervisors and co-workers. Supportive communication is a key element of social support [18], and active listening, or supervisors’ listening-centred communication, is a key feature of supportive leadership communication [19,20,21,22,23]. Supportive communication, through active-empathetic listening, may improve mutual understanding and reduce individuals’ and work teams’ uncertainty and help them feel as if they have control over their work [18]. 

However, in spite of a growing body of research on work engagement and social support, there is a need for a better understanding of the key feature of workplace social support—i.e., supervisor’s active-empathetic listening—and how it may influence work engagement. This study seeks to further the understanding of supervisors’ active-empathetic listening as an important part of workplace social support, one which can foster employee work engagement. To achieve this objective, we first review the existing literature on key concepts and previous findings on work engagement in relation to predictors such as social support. We then present our methods and the results of our investigation, before concluding with a discussion and recommendations for further research. 

## 2. Concepts and Prior Research

### 2.1. Well-Being at Work, Work Engagement, and Social Support 

Work-related well-being has been characterised by job satisfaction [24,25,26]. Further, it has been associated with the absence of mental strain—such as emotional exhaustion, reduced personal accomplishment, or a low self-evaluation of effectiveness at work—and the absence of cynicism or detachment from work [8,24,25,27]. Previous research, as mentioned before, emphasises that employees’ work engagement is an important predictor of well-being at work. 

In essence, work engagement determines how employees experience their work. A positive, fulfilling, and affective-motivational state of work-related well-being that is characterised by vigour, dedication, and absorption is most often considered the definition of work engagement (see, e.g., [1,3,4]). Vigour refers to the mental resilience of energetic employees while engaged in their work [1]. Dedication refers to high job accomplishment, a sense of significance, enthusiasm, and inspiration [13]. Absorption arises when individuals are fully concentrated on their job and find it difficult to disengage from what they are doing [1,5,13]. 

Work engagement is assumed to be the positive antipode of burnout [8,15,28]. According to Bakker et al. [1], vigour and dedication are considered direct opposites of exhaustion and cynicism. Exhaustion and cynicism are two of the three core symptoms of burnout. However, work engagement is characterised by energetic and dedicated employees who are enthusiastic, involved, and fully concentrating on their work [1]. Schaufeli and Bakker’s [8] findings indicated that burnout and work engagement are negatively related and that burnout is mainly predicted by job demands and a lack of job resources. Work engagement, on the other hand, is exclusively predicted by available job resources. Schaufeli and Bakker’s [8] results also confirmed prior findings that burnout is related to health problems and bad work-related well-being, as well as turnover intentions, whereas work engagement is related only to turnover intentions and job satisfaction. 

Several prior studies of work engagement refer to the job demand-resource (JD-R) model, with job resources and social support in focus (see, e.g., [2,5,6,8,13,17]). Orgambídez-Ramos and de Almeida [5] pointed out that, according to the JD-R model, work engagement functions as a mediator between job resources, organisational outcome, and employees’ well-being at work. Bakker and Demerouti [29] maintained that job resources refer to those physical, psychological, social, and organisational aspects of the job that might reduce the negative effects of high job demands (e.g., workload and unfavourable physical and mentally stressful work environment). The job resources identified in prior work engagement research are, e.g., job control, autonomy, performance feedback, social support, and job competence [1,2,15,30]. Orgambídez-Ramos and de Almeida [5] said that, among the job resources included in the JD-R model, social support is considered to be one of the most important resources mediating work engagement and well-being at work (see also, e.g., [3,7,17]). Karasek and Theorell [31] (p. 69) has defined workplace social support as the “overall levels of helpful social interaction available on the job from both co-workers and supervisors” that might affect the well-being of employees. Different types of workplace social support can involve offering concrete help and instrumental support, as well as advice on how to approach a problem, along with useful information dissemination and offering feedback and suggestions [32,33]. Jonsdottir et al. [34] pointed out that important types of social support in the workplace are offering advice, listening, and showing empathy or trust. 

Many scholars have concluded that job resources such as social support are an important antecedent and a salient predictor of work engagement and well-being at work. The findings of Hakanen et al. [13] showed that job resources such as job control, supervisory support, dissemination of information, and social climate are predictors of organisational commitment when mediated by work engagement. Simpson’s [10] findings determined interaction and open communication as significant antecedents of engaging employees and minimising the risk of them thinking of quitting their jobs. The findings of Pohl and Galletta [7] showed that when supervisors’ emotional support was high, the relationship between nurses’ (working at group level) work engagement and job satisfaction was stronger. Othman and Nasurdin [6] studied nurses’ working conditions and found that supervisor support was positively related to the nurses’ work engagement, whereas co-workers’ support had little or no effect on their work engagement. Mattson and Hall [18] also argued that the key feature of social support is communication, an important dimension of which is active-empathetic listening [20,21,23]. 

### 2.2. Supervisors’ Active-Empathetic Listening 

Jonsdottir and Fridriksdottir [21] pointed out that Mineyama et al. [23] used the term active listening in relation to supervisors’ listening-centred communication. Purdy [35] wrote about conscious and effective listening, which refers to active listening. Active listening is a way of listening to another person to gain a deeper understanding of the message and context. It is also the skill of responding effectively to the other person and the message with emotional intelligence [20,21,35,36].

According to both Drollinger et al. [37] and Brownell [20,38], there are several stages in the active-empathetic listening process. These can be described as, firstly, the sensing stage. The words are heard, and the listener implies that he/she is truly listening through the use of body language, gestures, and other forms of non-verbal acknowledgement. Secondly, there is the interpreting stage, where the listener evaluates the true meaning behind the received message. Last but not least, there is the responding stage, where the listener indicates to the others (those who speak) that the message has been received and informs them of how events will continue [21,36]. 

Research indicates that supervisors’ active-empathetic listening enhances good and supportive working conditions through open communication between the supervisor and subordinates, which is an important predictor of employees’ well-being [23]. Mineyama et al. [23] found that employees who had a supervisor with a higher active-empathetic listening score experienced higher job control. Further, Mineyama et al. [23] concluded that a supervisor’s active listening is an important factor in determining work-related stress among employees. This is in line with the findings of Ikemi et al. [39]. They found that employees who rated their supervisor as having a higher person-centred attitude, which is a core attitude for active-empathetic listening, had less depression and anxiety and were less fatigued (see, e.g., [36]). Lloyd et al. [22] concluded, from their results, that supervisors’ active-empathetic listening had positive effects on employees’ emotional condition as well as their loyalty to the organisation. Eisenberger [40] argued that employees that perceive their supervisor as being supportive believe that their supervisor has concern for their feelings and needs and cares about their well-being. 

To the best of our knowledge, this study is the first to extend the previous research literature by investigating the important dimension of supervisors’ supportive communication—i.e., active-empathetic listening—in relation to work engagement. We aim to analyse this relationship by seeking answers to the following research question: How is supervisors’ active-empathetic listening associated with employees’ work engagement—i.e., vigour, dedication and absorption? 

## 3. Methods 

### 3.1. Study Design and Participants 

This is a cross-sectional study that uses linear regression analysis to investigate the association between supervisors’ active-empathetic listening and employees’ work engagement. The participants were part of a panel maintained by the Social Science Research Institute in Iceland, and data gathering was conducted in cooperation with the institute. The panel is a representative sample of the Icelandic population and consists of people 18 years old or older. It was constituted using a random sample from the National Register of Iceland. Per the Icelandic Guidelines for Research Ethics [41], our research did not require approval from an institutional review board, as we were not collecting health data or working with vulnerable individuals. Informed consent was obtained from all the participants, and all data was anonymised.

Data gathering was conducted by an online survey (*N* = 1437) using a self-rated questionnaire with a response rate of 61% (*N* = 873). Our survey’s response rate was higher than the response rates that internet surveys generally have [42]. Additionally, the time-response method did not indicate response bias [43], and the same applied to socio-economic comparisons (age and gender). However, in our sample the participants were more educated than the average population. In the general population, 62.2% have finished and continued beyond compulsory education. In our sample, the comparable figure was 87.6%. The final sample (*N* = 548) for our analysis consisted of employed participants who finished the survey, with 273 males (49.9%) and 275 females (50.1%) meeting that criterion. Our exclusion criterion was therefore that participants needed to be employed. 

### 3.2. Procedure and a Pilot Study

To refine our research methods and measurements, a pilot study was first conducted in 2014. In the pilot study, a convenience sample (*N* = 159) was gathered through social media and used to refine measurements. The research assistant for the pilot study then used these data for his own Master’s thesis under the current authors’ supervision [44]. 

In the present study, employed participants were asked to rate their work engagement as well as the active listening skills of their immediate supervisor in an online survey. Prior studies have used the same method for data gathering [22,36,39]. However, it must be noted that this method does have the drawback of possibly introducing a halo effect into our data [45]. Although the presence of the halo effect might possibly influence our data, previous research has not found this to be the case, with the employees rating their supervisors’ active listening skills accurately [22]. Given our large representative sample, we opted for this approach. 

### 3.3. Measures

The participants answered both the active-empathetic listening scale as well as the Utrecht Work Engagement Scale (UWES). Additionally, we collected several individual-level demographic variables as controls. To validate our measurements, we reduced acquiescence by reverse-scoring some items. Additionally, using MV-marker variable methodology, we checked for common method variance and found no indication of common method bias in our data [46]. The Pearson correlation analysis indicated that there was no significant relationship between the marker variable and the active-empathic listening scale (*r*(451) = 0.07, *p* = 0.16).

#### 3.3.1. Active-Empathetic Listening Scale

Active-empathetic listening was measured using the eleven-item active-empathetic listening scale introduced by Drollinger et al. [37]. The original scale is a self-reporting scale but was adapted to an observer report scale to assess the perceived listening skills of the participants’ direct supervisor [47,48]. The participants were asked to note how much they agreed with each statement on a five-point Likert scale. Active-empathetic listening was therefore measured by statements such as: “My direct supervisor summarises points of agreement and disagreement when appropriate”. Active-empathetic listening scale (M = 3.39, SD = 0.88) had a Cronbach’s alpha coefficient of 0.96, which is higher than in previous studies (0.74–0.77). 

#### 3.3.2. UWES

To measure engagement, the UWES [49] was used. The original UWES uses seventeen statements, but to increase the response rate a shorter scale consisting of nine statements was used (α = 0.94) [50]. Many previous studies on work engagement have also used this scale, making comparisons with previous studies easier. According to previous empirical work, the UWES consists of three dimensions: vigour (α = 0.84), dedication (α = 0.87), and absorption (α = 0.82). For each statement, the participants rated each statement on a scale from 0 (never) to 6 (daily). Therefore, UWES was measured by phrases such as “I am enthusiastic about my job” and “At my job, I feel strong and vigorous”.

### 3.4. Data Analysis

The analysis of the data was performed with SPSS statistics 26 using multiple linear regression to test whether the supervisors’ listening skills were significantly related to work engagement. We found no indication that the linear regression assumptions were violated. To avoid omitted variable bias, several individual-level demographic variables were used as control variables in our regression analysis. The profile of the participants of this study is presented in Table 1.

### 3.5. Compliance with Ethical Standards

The authors declare that they comply with all the guidelines given by the Science Ethics Committee at the University of Iceland. Per the Icelandic Guidelines for Research Ethics, this research does not require approval from an institutional review board. Informed consent was obtained from all the participants and all the data were anonymised.

## 4. Results

In Table 2 and Table 3, we report the results from the multivariate regression models. The results in Table 2 indicate that our model explains 10% of the variance of work engagement (*R*^2^ = 0.10, *F*(6, 399) = 7.13, *p* < 0.01). We found that active-empathetic listening had a significant positive relationship with work engagement when controlling for individual demographic factors. As can be seen in Table 2, each one-unit increase in active-empathetic listening is associated with a 0.235-unit increase in work engagement, holding all other independent variables constant.

As discussed earlier, the three dimensions of work engagement are vigour, dedication, and absorption [1]. In Table 3, we report the results of our analysis of the relationship between the three work engagement dimensions—i.e., vigour, dedication, and absorption—and the supervisors’ active-empathetic listening. For vigour, the results indicate a significant relationship to active-empathetic listening, with our model explaining 4% of the variance (*R*^2^ = 0.04, *F*(6, 399) = 3.94, *p* < 0.01). As can be seen in Table 3, each one-unit increase in active-empathetic listening is associated with a 0.179-unit increase in the vigour dimension of work engagement, holding all other independent variables constant. When examining dedication, the results indicate a significant relationship with active-empathetic listening. The model explained 12% of the variance (*R*^2^ = 0.12, *F*(6, 399) = 9.88, *p* < 0.01) in dedication. In our model, each unit increase in active-empathetic listening is associated with a 0.333-unit increase in the dedication dimension of work engagement. From Table 3, we observe that Model 3 for the absorption dimension of work engagement explains 5% of the variance (*R*^2^ = 0.05, *F*(6, 399) = 4.687, *p* < 0.01), with each unit increase in active-empathetic listening being associated with a 0.196-unit increase in the absorption dimension of work engagement. Overall, our results indicate that active-empathetic listening contributes the most to the dedication dimension of work engagement, followed by absorption and vigour, respectively.

In line with the analysis by Mineyama et al. [23], we also split the participants into groups with high and low active listening supervisors. We made the split according to the median score on the active-empathic listening scale (*Mdn* = 3.45) and compared the factors of work engagement between the groups, while also using Cohen’s effect size benchmark [51] to determine the magnitude of differences between the group means. 

In Table 4, we explore the differences between employees who perceived their supervisors to be high- versus low-skilled active-empathetic listeners and their effect on the symptoms of work engagement. We conducted a series of *t*-tests to compare the groups. Employees with high-scoring active-empathetic listening supervisors reported significantly higher vigour (M = 5.70) scores than employees with low-scoring (M = 5.40) active-empathetic listening supervisors, *t* (446) = −2.77, *p* < 0.01, *d* = 0.26. For the dedication symptom, it was found that high-skilled active-empathetic listening supervisors had employees with significantly higher dedication (M = 5.79) than low-skilled active-empathetic listening supervisors (M = 5.33), *t* (441) = −3.87, *p* < 0.01, *d* = 0.37. However, for absorption, we did not find a significant difference between the high-skilled active-empathetic listening supervisors (M = 5.55) and the low-skilled active-empathetic listening supervisors (M = 5.33), *t* (447) = 1.77, *p* > 0.05, *d* = 0.17. 

## 5. Discussion

The overall objective of this study is to analyse the role of supervisors’ active-empathetic listening in employees’ engagement at work. We are keen to find out whether there are any differences in the effects of supervisors’ active-empathetic listening on the three dimensions—i.e., employees’ vigour, dedication, and absorption.

The findings of this research are in line with several studies that highlight the importance of job resources, especially supervisor social support, as a salient predictor of work engagement [5,6,7,10,13,36]. However, as Lam [52] argued, the concept of social support has a complex meaning, as it is subject to a series of interactions and processes that cover different working conditions. Scholars who investigated social support in relation to work engagement have referred to social support in the workplace as (group) discussion, emotional support, mentoring, helpful social interaction on the job, or even the creation of a culture characterised by trust and positive relationships [5,6,7,17]. We claim that such arguments regarding social support are too generalised and make practical organisational interventions difficult. 

In our study, we lean on definitions of social support as communication nexus [18] and on Albrecht and Adelman’s [53] definition of social support as “verbal and nonverbal communication between recipients and providers that reduces uncertainty about the situation, the self, the other, or the relationship, and functions to enhance a perception of personal control in one’s life experience” (p. 19). Active-empathetic listening is a critical dimension in managerial communication [21] that is often taken for granted [54]. Taking a stand in these theoretical arguments [53,54], we argue that one important dimension of workplace social support as a communication nexus is supervisors’ active-empathetic listening. We argue that our study is the first to investigate this as a predictor and an antecedent of employees’ work engagement. 

Overall, our results show that a significant positive relationship exists between supervisors’ active-empathetic listening and their employees’ work engagement. Delving further into the results, we find indications of this positive relationship applying to all three dimensions of work engagement. However, a supervisor’s active listening does not seem to affect all three dimensions equally, with dedication being the most affected, followed by vigour and finally the absorption dimension. 

In our study, the employees who perceived supervisors as high-scoring active-empathetic listeners reported significantly higher dedication scores compared to those employees who perceived supervisors to be low-scoring active-empathetic listeners. This result indicates that supervisors’ active-empathetic listening is a potential work-related predictor of employees’ higher professional and personal work accomplishment in their work. It suggests that supervisors who truly, actively, and empathetically listen boost their employees’ effectiveness and enthusiasm and enhance their involvement in work. By active-empathetic listening, they give employees an opportunity to speak and deliver a message and then strive to comprehend and interpret the message (e.g., read the body language and “listen to the song beneath the words” [55] (p. 64)) and, last but not the least, respond to the message. Its strong effect on dedication is important, as it reduces uncertainty, develops competence, and enhances the self-esteem of employees. This, in return, might result in job satisfaction, better job control, and overall perceptions of good working conditions. 

Our findings also suggest that high-scoring active-empathetic listening supervisors are antecedents of vigour—i.e., employees feeling that they possess more emotional energy and physical strength at work [56]. Vigour is of importance, as Shirom [56] mentioned, because it has been found to positively predict employees’ good health (lower risk of exhaustion) and proactive behaviour at work. Further, the results imply that supervisors who listen consciously and actively positively affect their employees’ cynicism or low concentration at work. A noteworthy finding is, however, that for the absorption dimension, we only find a marginally significant difference between high- and low-scoring active-empathetic listening supervisors. 

Our results showing employees’ higher dedication at work and higher vigour as being correlated with their supervisors’ active-empathetic listening are interesting in the light of prior empirical findings that indicate that perceptions of high-scoring supervisors’ active listening are positively related to employees’ reports of working conditions with open communication, job control, job satisfaction, and less anxiety or mental distress [22,23,36,39]. 

### Limitations and Further Research

As with any research, care must be taken when interpreting our findings. Firstly, a limiting factor for our research data is the subjective nature of our data. That is, the listening measure that we used reflects employees’ subjective perception of their supervisors’ active-empathetic listening. Having a more objective measure would, of course, be ideal. Since our data are a representative sample of the Icelandic population, remedying this limitation is difficult. Although this limitation is problematic for inference, prior research suggests that employees’ perceptions of their supervisors’ active listening reflect supervisors’ self-ratings [22], which strengthens our result.

Secondly, the data collected are both cross-sectional and self-reported in nature. Therefore, although we found a clear relationship between supervisors’ active listening and employees’ work engagement and its sub-dimensions, these results must be further validated with additional data gathering and analysis. An interesting research endeavour in this direction would be to examine whether a causal relationship between active-empathic listening and work engagement can be established. This could be managed through a field experimental design that has proven valuable in other areas of management research [57,58].

Additionally, there is a need for further investigation into the roles of both supervisors’ active-empathetic listening and work engagement and how these relate to dimensions of work-related quality of life, e.g., work-related stress, job control and job satisfaction. Mixed-methods research could shed further light on the working conditions and employees’ perceptions of the two phenomena by adding information about the human experience (qualitative data) to the study. 

## 6. Conclusions

In today’s turbulent and demanding work environment where the focus is on employees’ well-being at work (which involves, e.g., job satisfaction and the absence of negative work stress), our research project is quite important. Based on what we know from reviewing the literature on job resources, especially social support, work engagement, work-related well-being, and organisational benefits, we argue that work engagement is critically important in today’s organisations. Further, we conclude from our investigation, that supervisors’ active-empathetic listening is an important but under-researched antecedent of employees’ engagement at work. Our findings suggest that supervisors who are skilled in active-empathetic listening positively affect their employees’ accomplishments, enthusiasm, involvement at work, and emotional energy. The results indicate that workplaces and management need to pay more attention to supportive leadership which considers conscious and active listening-centred communication as highly significant for employees’ perception of good working conditions and, subsequently, their work engagement. 

Although our results are only the first step in examining the importance of supervisors’ active listening for employees’ work engagement, we do feel that some practical implications are in order. Therefore, we suggest that organisations experiment in training their supervisors in active-empathetic listening as a part of a broader strategy to promote supportive leadership and good communication practice and, in turn, increase employees’ work engagement.

## Figures and Tables

**Table 1:** Descriptive statistics.

	Mean	SD	*N*
Dependent variables			
Work Engagement	5.53	1.12	490
Vigour	5.56	1.13	488
Dedication	5.56	1.26	490
Absorption	5.45	1.32	490
Independent variable			
Active-empathetic listening (AEL)	3.39	0.88	453
Control variables			
Gender	1.50	0.50	548
Age	42.07	14.23	548
Income	0.34	0.48	430
Education	0.61	0.49	470
Children	0.47	0.50	475

**Table 2:** Work engagement and supervisors’ active-empathetic listening.

	*B*	*SE*	β
*(Constant)*	3.996 ***	0.317	
AEL	0.235	0.059	0.193 ***
Gender	0.236	0.108	0.110 *
Age	0.012	0.004	0.160 **
Income	0.374	0.121	0.165 **
Education	−0.226	0.111	−0.103 *
Children	−0.145	0.107	−0.068
*F*		7.134	
*Adjusted R* ^2^		0.097	

* *p* < 0.05 ** *p* < 0.01 *** *p* < 0.001.

**Table 3:** The dimensions of work engagement and supervisors’ active-empathetic listening.

	Model 1: Vigour			Model 2: Dedication			Model 3: Absorption		
	*B*	*SE*	β	*B*	*SE*	β	*B*	*SE*	β
*(Constant)*	4.528 ***	0.335		3.458 ***	0.360		3.997 ***	0.366	
AEL	0.179	0.062	0.142 **	0.333	0.067	0.236 ***	0.196	0.068	0.141 **
Gender	0.099	0.114	0.045	0.298	0.122	0.120 *	0.282	0.124	0.116 *
Age	0.008	0.004	0.104	0.015	0.004	0.176 **	0.013	0.004	0.158 **
Income	0.370	0.128	0.158 **	0.473	0.137	0.181**	0.293	0.139	0.114 *
Education	−0.184	0.118	−0.081	−0.205	0.127	−0.081	−0.372	0.129	−0.109 *
Children	−0.090	0.113	−0.041	−0.240	0.122	−0.097 *	−0.133	0.124	−0.054
*F*		3.942			9.881			4.870	
*Adjusted R* ^2^		0.042			0.116			0.054	

* *p* < 0.05 ** *p* < 0.01 *** *p* < 0.001.

**Table 4:** Work engagement dimensions and high/low-skilled active-empathetic listening.

	Active-Empathetic Listening					
	High (*n* = 217)		Low (*n* = 231)			
	Mean	SD	Mean	SD	*p*	*d*
Work Engagement						
Vigour	5.70	1.10	5.40	1.16	***	0.26
Dedication	5.79	1.21	5.33	1.28	***	0.37
Absorption	5.55	1.40	5.33	1.24	*	0.17

* *p*< 0.10; *** *p* < 0.01.
